# Promoting state health department evidence-based cancer and chronic disease prevention: a multi-phase dissemination study with a cluster randomized trial component

**DOI:** 10.1186/1748-5908-8-141

**Published:** 2013-12-13

**Authors:** Peg Allen, Sonia Sequeira, Rebekah R Jacob, Adriano Akira Ferreira Hino, Katherine A Stamatakis, Jenine K Harris, Lindsay Elliott, Jon F Kerner, Ellen Jones, Maureen Dobbins, Elizabeth A Baker, Ross C Brownson

**Affiliations:** 1 Prevention Research Center in St. Louis, Brown School, Washington University in St. Louis, 621 Skinker Blvd., St. Louis, MO 63130-4838, USA; 2 Department of Physical Education, Federal University of Parana, Curitiba Parana, Brazil; 3 Department of Physical Education, School of Health and Biosciences, Pontifícia Universidade Católica do Paraná, Curitiba, Parana, Brazil; 4 Department of Epidemiology and Prevention Research Center in St. Louis, College for Public Health & Social Justice, Saint Louis University, 3545 Lafayette Ave, St. Louis, MO 63104, USA; 5 Population Health and Knowledge Management, Canadian Partnership Against Cancer, Toronto, ON M5J 2P1, Canada; 6 School of Health Related Professions, University of Mississippi Medical Center, Jackson, MS 39216, USA; 7 National Collaborating Centre for Methods and Tools and Health Evidence, McMaster University, Hamilton, Ontario, Canada; 8 Prevention Research Center in St. Louis, College for Public Health & Social Justice, Saint Louis University, 3545 Lafayette Ave, St. Louis, MO 63104, USA; 9 Division of Public Health Sciences and Siteman Cancer Center, Washington University School of Medicine; Washington University in St. Louis, St Louis, MO 63130, USA

**Keywords:** Information dissemination, Innovation diffusion, Dissemination research, Public health workforce, Chronic disease prevention, Cancer prevention and control, Evidence-based public health, Public health accreditation

## Abstract

**Background:**

Cancer and other chronic diseases reduce quality and length of life and productivity, and represent a significant financial burden to society. Evidence-based public health approaches to prevent cancer and other chronic diseases have been identified in recent decades and have the potential for high impact. Yet, barriers to implement prevention approaches persist as a result of multiple factors including lack of organizational support, limited resources, competing emerging priorities and crises, and limited skill among the public health workforce. The purpose of this study is to learn how best to promote the adoption of evidence based public health practice related to chronic disease prevention.

**Methods/design:**

This paper describes the methods for a multi-phase dissemination study with a cluster randomized trial component that will evaluate the dissemination of public health knowledge about evidence-based prevention of cancer and other chronic diseases. Phase one involves development of measures of practitioner views on and organizational supports for evidence-based public health and data collection using a national online survey involving state health department chronic disease practitioners. In phase two, a cluster randomized trial design will be conducted to test receptivity and usefulness of dissemination strategies directed toward state health department chronic disease practitioners to enhance capacity and organizational support for evidence-based chronic disease prevention. Twelve state health department chronic disease units will be randomly selected and assigned to intervention or control. State health department staff and the university-based study team will jointly identify, refine, and select dissemination strategies within intervention units. Intervention (dissemination) strategies may include multi-day in-person training workshops, electronic information exchange modalities, and remote technical assistance. Evaluation methods include pre-post surveys, structured qualitative phone interviews, and abstraction of state-level chronic disease prevention program plans and progress reports.

**Trial registration:**

clinicaltrials.gov: NCT01978054.

## Background

The burden to individuals, families, communities, and society from tobacco use, poor nutrition, inadequate physical activity, obesity, and related cancers, cardiovascular diseases, and diabetes is staggering and has been well-documented [1]. With the aging of the population, growth in healthcare costs to manage chronic diseases threatens state and national economies. In 2010, overall costs for cancer alone were over $124 billion [2]. Multiple chronic diseases are common, with 21% of those aged 45 – 64 years old in the U.S. having two or more chronic diseases, and 62% of those aged 65 years and older [3,4]. Low income and minority populations carry an excess burden due to early onset, later diagnosis, and poorer disease management outcomes [5-7]. Health-enhancing behaviors, including physical activity, healthy eating, and avoiding tobacco, can delay or prevent chronic disease [8-12]. In addition, management of existing conditions through health-enhancing behaviors has been found to improve quality of life and reduce healthcare costs [13].

In the past two decades, environmental and policy approaches to prevent cancer and other chronic diseases have been identified that provide the potential to reach entire communities and populations statewide [14-18]. Because tobacco use, physical activity, and poor nutrition are major risk factors not only for some cancers, but also for cardiovascular diseases and other chronic conditions [19], this study addresses prevention of multiple chronic diseases including cancer (here after referred to as chronic disease prevention) (see Table 1). While effective interventions in the areas of tobacco, physical activity, and cancer screening are well-established, more recent evidence is building for effective nutrition interventions [20-27]. Despite great progress in identifying effective interventions, challenges to implementing these interventions remain. These include reaching large populations and addressing barriers associated with implementing and adapting interventions across multiple settings and populations, including low income and minority populations [28].

**Table 1:** Interrelationships among various chronic diseases and modifiable risk factors, United States

	**Cardiovascular disease**	**Cancer**	**Chronic lung disease**	**Diabetes**	**Cirrhosis**	**Musculoskeletal diseases**	**Neurologic disorders**
Tobacco use	**+**	**+**	**+**			**+**	**+**
Alcohol use	**+**	**+**			**+**	**+**	**+**
High cholesterol	**+**						
High blood pressure	**+**						**+**
Diet	**+**	**+**		**+**		**+**	**?**
Physical inactivity	**+**	**+**		**+**		**+**	**+**
Obesity	**+**	**+**		**+**		**+**	**+**
Stress	**+**	**?**					
Environmental tobacco smoke	**+**	**+**	**+**				**?**
Occupation	**+**	**+**	**+**		**?**	**+**	**?**
Pollution	**+**	**+**	**+**				**+**
Low socioeconomic status	**+**	**+**	**+**	**+**	**+**	**+**	

Reprinted with permission from Remington RL, Brownson RC, Wegner MV, eds. Chronic Disease Epidemiology and Control, Third Edition. 2010: American Public Health Association, Washington, DC [19].

An additional barrier to evidence-based chronic disease prevention is the challenge of keeping up-to-date the knowledge and skills of the public health workforce. Even today, only a small portion of the public health workforce has formal academic training in public health [19,29,30]. Evidence-based policies and programs (EBPPs) for chronic disease prevention are complex and implemented across multiple settings and levels of society. This, compounded by high staff turnover in public health agencies, adds to the challenge of maintaining knowledge and skill to practice in an evidence-informed way [29].

Evidence-based public health requires knowledge of processes as well as specific intervention evidence content knowledge and a complex set of skills. Such process knowledge is a key part of evidence-based decision making (EBDM), which involves the integration of science-based interventions with community preferences to improve the health of populations [31]. EBDM involves multiple processes, including making decisions based on the best available scientific or rigorous program evaluation evidence, applying program planning and quality improvement frameworks, engaging the community in assessment and decision-making, adapting and implementing EBPPs for specific populations or settings, and conducting sound evaluation [32-34]. To select and implement EBPPS with diverse populations and settings, advanced knowledge and skill is needed in intervention adaptation and implementation processes.

Therefore, in order to increase use of EBDM and EBPPs it is important to determine how best to disseminate public health knowledge and evolving scientific evidence to build public health agency workforce capacity and organizational support for evidence-based chronic disease prevention. The National Cancer Institute (NCI) has acknowledged the need for more effective dissemination by making effective dissemination and application of cancer research findings a major theme in its strategic plan [35]. Thus, public health agency level interventions where dissemination strategies can be evaluated at the organizational (or cluster) level are necessary.

The goals of this multi-phase dissemination study are to determine how best to increase individual awareness and capacity of state-level public health practitioners to apply EBDM processes and EBPPs for prevention of cancer and other chronic diseases; increase agency and individual level application of EBDM processes to prevent cancer and other chronic diseases in applicable work units within state health departments; and increase agency level promotion of effective approaches and EBPPs with local public health agencies and partnering organizations.

## Methods/design

### Study design

This is a multi-phase dissemination study funded by the NCI to learn which dissemination strategies best support uptake and application of EBDM processes among state health department practitioners and their key partners that work in cancer and other chronic disease prevention program areas. This multi-phase study is guided by an international advisory group of university-based researchers, former public health practitioners from state health departments, as well as collaborators from Canada with experience in dissemination research. The two study phases overlap and complement each other. Phase one involves development and testing of a self-report survey and archival report abstraction instrument. Phase one also includes collection of self-report data from a national representative sample of state health department practitioners working in chronic disease prevention. Phase two is a group randomized evaluation study. Phase two involves stratified random selection of six dissemination and six pair-matched comparison state health departments to test acceptance and usefulness of the identified dissemination strategies to state-level practitioners working in chronic disease prevention. Dissemination strategies may include training in evidence-based public health, technical assistance, and provision of brief user-friendly evidence summaries. Study collaborators include the National Association of Chronic Disease Directors (NACDD) and the Centers for Disease Control and Prevention (CDC) Division of Cancer Prevention and Control, with collaboration with other units at CDC as well. The study was approved by the institutional review board of Washington University in St. Louis. Some aspects of phase one have been completed, while phase two is in the planning phase. Phase two is registered as a cluster randomized trial (clinicaltrials.gov NCT01978054).

**Figure 1:**
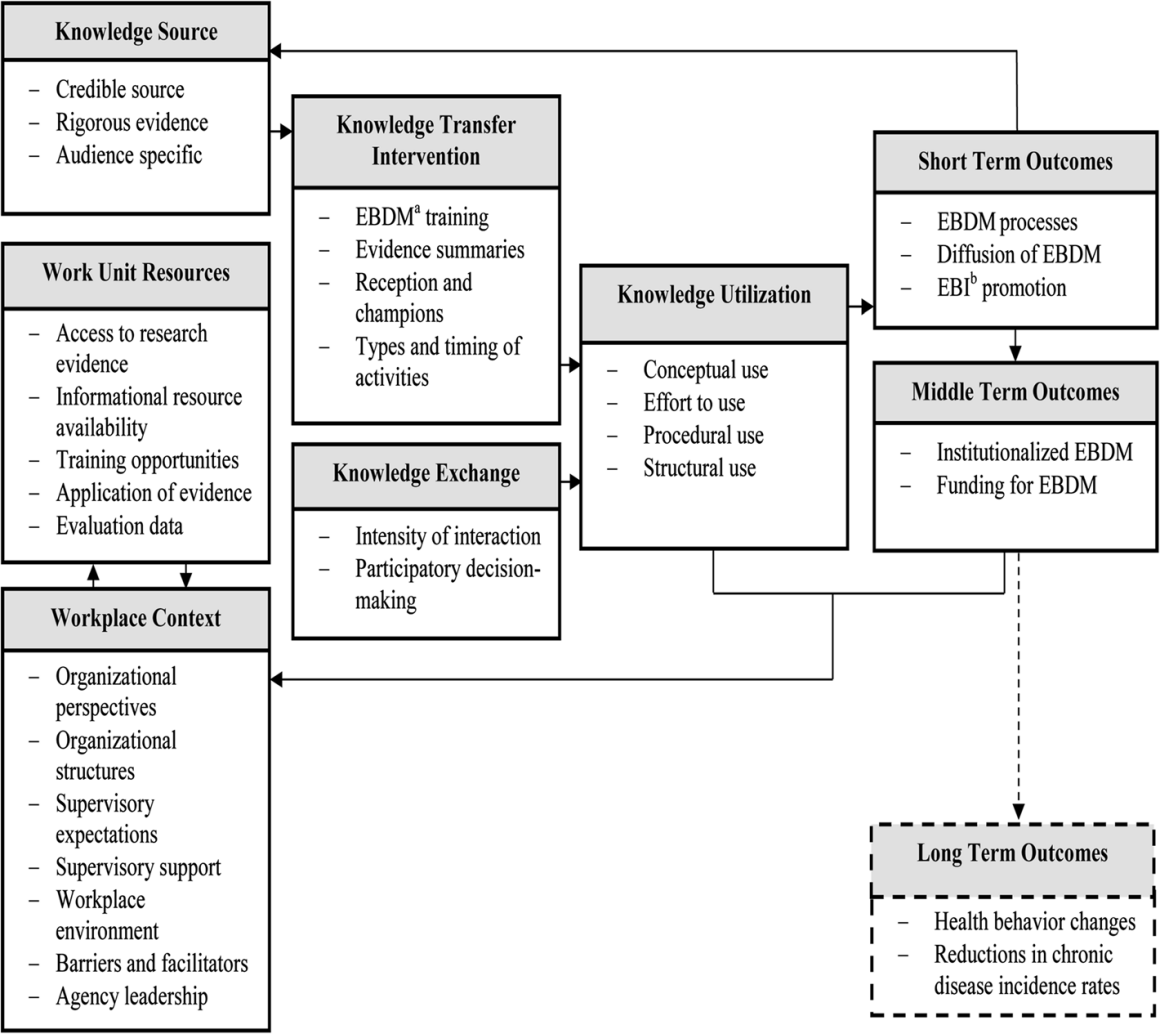
**Conceptual framework for dissemination of evidence based public health.** Framework adapted from: Kramer DM, Cole DC. Sci Commun. 2003; 25(1):56-82 [36]. Kramer DM, Cole DC, Leithwood K. B Sci Technol Soc. 2004; 24(4):316-330 [37]. Kramer DM, Wells RP, Carlan N, Aversa T, Bigelow PP, Dixon SM, McMillan K. JOSE. 2013; 19(1):41-62 [38].

The dissemination conceptual framework for the study is depicted in Figure 1. It is adapted with permission from Kramer and Cole’s [36]. Conceptual Framework for Research Knowledge Transfer and Utilization [37,38]. The study model is also informed by Diffusion of Innovations [39] and Institutional Theory [40-42]. Dissemination in this study is the process of enhancing the capacity of the target audience of state-level practitioners to apply EBDM processes to promote statewide and local planning, adaptation, implementation, and evaluation of specific EBPPs for chronic disease prevention [43]. The workplace context is hypothesized as a key determinant of how knowledge is received, used, and incorporated into the organization’s usual day-to-day operations. In knowledge transfer and knowledge exchange, there is a flow of information that affects not only the target audience of practitioners but also the researchers. Researchers use the terms knowledge translation and exchange or knowledge exchange to denote an interactive process in which practitioners and researchers together problem solve how to apply research knowledge in specific contexts [36,37,44-48]. As in this study’s framework (Figure 1), some researchers make a distinction between knowledge transfer that is largely unidirectional from researcher to practitioner and knowledge transfer and exchange that involves a social interactive process dependent on the quality of researcher-practitioner relationships [45,46]. In this study, researchers will learn from practitioners about key contexts that affect application of research knowledge, such as organizational climate and political influences. Practitioners will learn knowledge, skills, and evidence-based organizational practices from researchers. Skill development may address common public health workforce skill gaps, such as use of data including economic data for planning and evaluation, interpretation of intervention research findings, collaboration across disciplines for environmental changes, communication of evidence to policy-makers, and documentation of use of evidence-based approaches [49]. Together, practitioners and researchers will determine how best to enhance modifiable contextual elements to support evidence-based state level chronic disease prevention.

**Figure 2:**
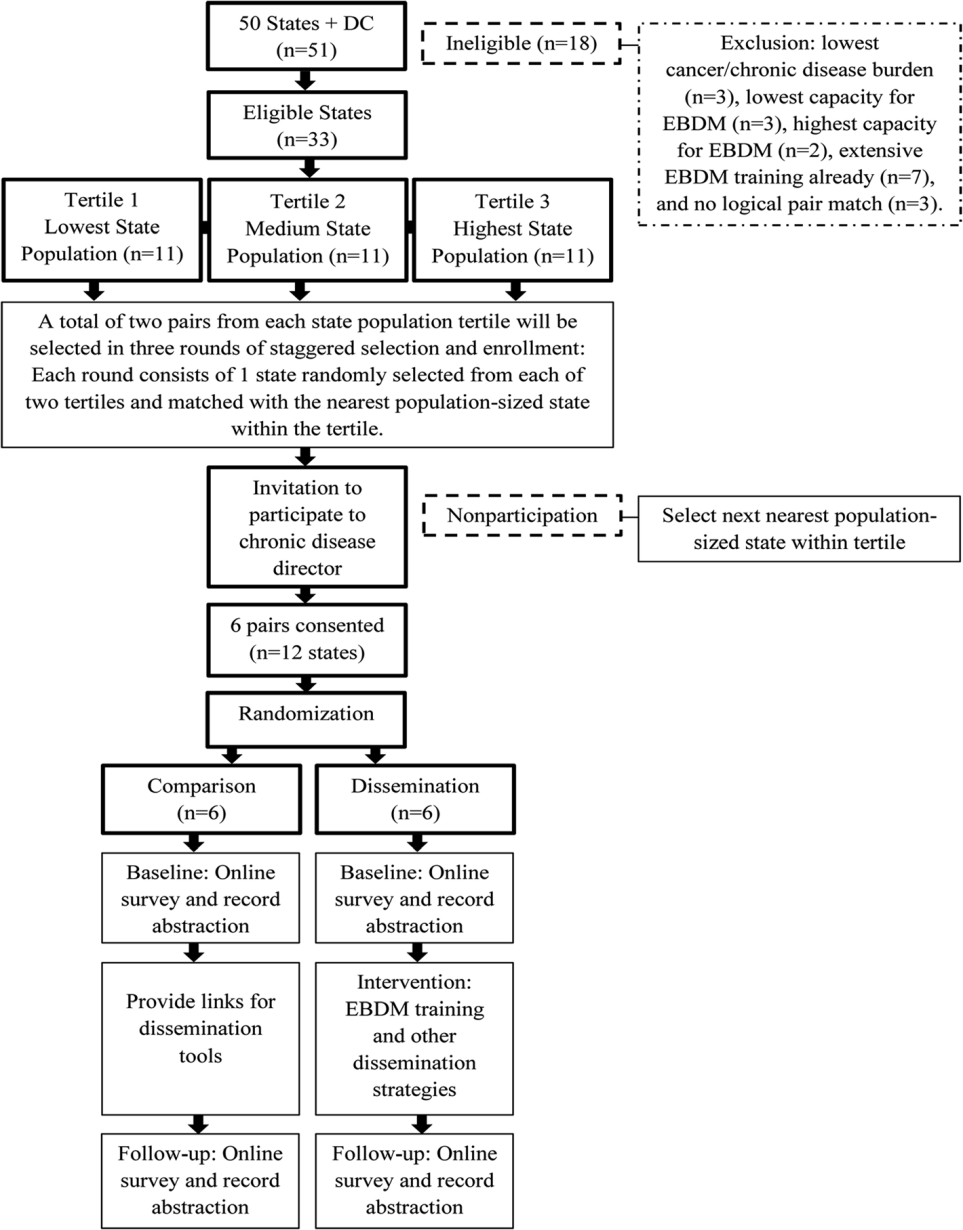
State health department selection process for phase two.

The study team will work in partnership with state-level practitioners from the dissemination states to develop user-friendly evidence materials and EBDM trainings; assess organizational and other factors that influence acceptance of EBDM; support state-level practitioner application of EBDM processes to enhance evidence-based chronic disease prevention; and design strategies to embed EBDM processes within ongoing practices. Evaluation will include process evaluation in dissemination states, and pre-post evaluation in dissemination and comparison states [38,43]. (See Figure 2).

### Study audience

The target audience is state health department practitioners working in comprehensive cancer prevention and control, cancer screening, tobacco control, physical activity, nutrition, obesity prevention, school health, diabetes prevention, and cardiovascular health. State health departments typically provide funding, informational resources, and guidance for the implementation of EBPPs by state and local coalitions, local public health departments, and other agencies more than directly implementing policies and programs. The study involves the provision of several EBDM dissemination strategies, identified by the study team targeted to participating state health department chronic disease units [35,43].

### Phase one: development of measures

In phase one the study team has developed and tested a survey instrument and collected self-report survey data nationally. In addition, the study team will develop a tool to abstract archival state health department plans and progress reports as an objective source of data on planning and implementation of EBPPs in chronic disease prevention.

### Survey instrument

The main objective of the self-report online survey are to obtain a national snapshot of practitioner views on EBDM, training and informational needs for EBDM, organizational support for EBDM, barriers to the application of EBDM, and EBPP implementation among state health department mid-level program managers and staff working in chronic disease prevention across the United States.

### Measures

The survey was developed from previous research conducted by Dr. Brownson *et al.*[32,49], a literature review [50], and five rounds of study advisory group input from August-November 2012. The 68-item survey contains eight sections and was designed for completion in about 15 minutes. Table 2 describes the survey domains and types of items included in the survey, as well as their sources. A state-level global score for EBPPs being implemented in the state was developed for use as either a dependent or independent variable. From the EBDM skill importance and availability scales, a gap score subtracts perceived availability from perceived importance [49]. Two items on perceived benefits and challenges of coordinated chronic disease prevention were pilot tested by 10 Missouri state department of health coordinated chronic disease committee members and revised based on feedback received. The survey instrument was programmed in Qualtrics online survey software and underwent several periods of trial and refinement internally with research staff prior to cognitive response and reliability testing.

**Table 2:** Survey measures

**Survey section**	**Number of items**	**Type of variables**	**Subscales or sample items**	**Item sources**
Biographical	14	Yes/no, number of years, check one, check all that apply	Position, program area	Jacobs 2010
			Years at state health department	Reis (in press)
			Years in public health	
EBIs implemented (selection pattern based on program area)	Varied by topic asked	Yes/no/don’t know	Asked 1 to 2 of 6 topics: cancer screening, skin cancer prevention, tobacco, physical activity, nutrition, school health	Community guide
				Nutrition systematic reviews
Your views on EBPPs	9	Likert 7-point	I can effectively communicate information on evidence-based interventions to elected officials.	Jacobs 2010
				Reis (in press)
EBDM definitions and incentives	2	Rank top 3	Which of the following would most encourage you to utilize EBDM?	Jacobs 2012
				Reis (in press)
Importance and availability of EBDM elements	20	Likert 11-point (0-10)	Importance (10 items	Jacobs 2012
			Availability (10 items)	Reis (in press)
Use of EBDM	1	Likert 7-point	I use EBDM in my work	New
Workplace context	17	Likert 7-point	Supervisory support and expectations (3 items)	Brownson 2012
				Reis (in press)
			Work unit resources (5 items)	Stamatakis 2012
			Work unit knowledge exchange (2 items)	
			Work unit evaluation (3 items)	
			Agency leadership (2 items)	
Use of informational evidence resources	5	Yes/no, how often	• Use of community guide	Jacobs 2012
			• What methods allow you to learn about the current findings in public health research? (Rank top 3)	Reis (in press)
		Rank top 3		
		Check all that apply		
Coordination of chronic disease programs	2	Rank top 3	Perceived benefits	New
			Perceived challenges	

### Survey instrument testing

#### Cognitive response testing

Items from the instrument were revised through cognitive response testing, which has been shown to improve survey development [51]. Eleven former state health department chronic disease directors or program managers identified by a partner organization completed hour long interviews in December 2012, in which they reviewed the survey instrument with a research assistant and project manager. Participants provided feedback about what they thought the questions were asking, question wording that might be unclear to others, and questions that were clear but still difficult to answer. Participants also provided additional response options on a number of items. Interview participants were offered a $40 Amazon.com gift card for completion of cognitive response testing. Recorded interviews were reviewed to identify themes that occurred in two or more interviews and reviewed by the study advisory group who refined question wording.

### Reliability test-retest

We randomly selected 150 practitioners from contact lists collected from NACDD, CDC, and the Tobacco Technical Assistance Consortium. The 106 respondents that completed the survey the first time were each emailed an invitation to take the survey again within 14 to 24 days after their initial survey. Efforts were made to distribute the sample across states and program areas and all 50 states were represented in the reliability sample. Replacement sampling was done as needed to get 150 eligible invitees. Of the 150 eligible practitioners invited, 106 (70.7%) completed test one, and 75 completed test two (70.8% of test one). Respondents completed the second survey 10 to 30 days after the first survey. Among those that kept the online survey open less than 40 minutes, the median time to complete the survey was 18.5 minutes, with a mean time of 19.4 (SD = 7.3) minutes.

Test-retest statistical analyses included calculating intra-class correlation coefficients (ICC) for Likert scale items, and percent agreement and Cohen’s kappa statistic for dichotomized Likert-scale items (strongly agree and agree vs. other responses) [52,53]. To test internal consistency of the domain and influence of individual items on a domain, for each continuous variable, the Cronbach’s alpha was calculated. For ranking items, the percent agreement of the three items chosen in the top three in Test1 and Test2 was calculated. Landis and Koch [52] kappa categories of almost perfect (1.0 – 0.8), substantial (0.8 – 0.6), moderate (0.6 – 0.4), fair (0.4 – 0.2), and low (0.2 – 0.0) were used as qualifiers for interpretation of results. For ICCs and percent agreement, >0.70 were considered desirable and >0.80 were best [53]. Test-retest results showed that overall the percent agreements were typically ≥0.70 and ICCs, the appropriate statistic for most sections and items, were mostly ≥0.70. The majority of kappa coefficients were in the moderate range (0.40 – 0.60). Most of the scales showed adequate internal consistency (Cronbach’s alpha ≥0.70). Two questions were deleted after review of test-retest results and the wording of three items was modified. Because the survey was only slightly modified, test one completed surveys will be combined with the full survey sample data described below for nearly all items.

### Survey participant recruitment

The study team created a list of eligible individuals through exhaustive searching of US state health department websites and updated lists from NACDD and CDC. State health department practitioners working in primary and secondary cancer prevention and screening, physical activity, nutrition, tobacco, obesity, diabetes, cardiovascular health, healthy aging, and general chronic disease prevention in the US or a US territory were invited to participate in the survey. State health department employees of all ages, genders and educational backgrounds were included. Administrative assistant staff members were excluded. Invitations containing information on the survey and a survey link were emailed to state health practitioners in March 2013. Pre-invitations informing survey respondents about the purpose of the study were sent one week prior to invitations. Initial non-respondents received two follow-up calls and three email reminders, which resulted in a response rate of 75.5%. Respondents were offered an optional $20 Amazon.com gift card for completion of the survey.

National survey data collection resulted in a total of 923 completed surveys from state health department employees in all 50 states, the District of Columbia, and five of the eight US territories. Of the 1,443 invited into the survey, 221 were ineligible because they no longer worked at a state health department, were on an extended leave of absence, or now worked outside of chronic disease prevention. Of the 1,222 eligible invitees, 923 completed the survey for a response rate of 75.5%. The 19 surveys from health department staff from the U.S. territories will be excluded from initial analyses, because three of the eight territories did not participate, and only 36.5% (19 of 52) eligible invitees from the territories completed the survey. Therefore, a total of 904 completed surveys will be included in data analyses. Among the 50 states, the response rate was 77.3%.

### Program record review tool development

Phase one includes development of an abstract tool and codebook for archival record abstraction. The purpose of abstracting state health department plans and reports for various program areas in chronic disease prevention is to corroborate with and expand on self-report information on EBPPs being planned and implemented in chronic disease prevention in the twelve participating states. The study team will abstract health department progress reports and plans, as well as statewide coalition strategic plans, in cancer prevention and control, tobacco control, obesity prevention, physical activity, nutrition, cardiovascular health, and diabetes before and after dissemination strategies are applied. This will provide an objective gage of EBPP uptake before and after dissemination strategies are applied. The record abstraction tool will also be made available to other users online upon finalization. The Community Guide [54] will be the basis for EBPP inclusion on the tool for most program areas, while systematic reviews not yet incorporated into The Community Guide will be utilized to identify evidence-based nutrition EBPPs. To draft the record abstraction tool and codebook, the study team initially abstracted 32 state health department plans and program reports from 10 states. Additional interventions in updated Community Guide systematic reviews will be added as they become available.

Study staff then documented types of plans and reports publicly available from state health department websites in a random sample of six states. Publicly available information contained mostly plans for future strategies rather than information on what was currently being implemented. Of 84 documents found, 57 contained future plans only, and 27 contained future plans plus some information about implementation of current plans. From these findings along with professional consult, phase two’s abstraction form will be completed using state progress reports from the participating chronic disease prevention programs.

### Phase two: dissemination with state health departments

#### Overview

Phase two will be a paired, cluster randomized evaluation study to determine effective ways to disseminate public health knowledge about EBDM and chronic disease prevention EBPPs with mid- to senior-level state health department employees working in prevention of cancer and other chronic diseases. Clusters are state health department chronic disease units (hereafter called states) made up of their respective individual employees. There will be two parallel study arms with six dissemination (intervention arm) and six pair-matched comparison (control arm) states will be randomly selected from the 33 eligible states (see Figure 2) and invited to participate in a staggered enrollment schedule in years two and three of the study. The main purpose of the dissemination strategies is to build capacity and to explore optimal ways to package information for timeliness, relevance, and usefulness to public health practitioners. Participating dissemination states will help develop and choose three to five dissemination strategies they prefer for their state health department chronic disease units to receive. Dissemination strategies may include training in EBDM targeted to priority risk factors and program areas, issue briefs with user-friendly evidence summaries, targeted messaging, and information on ways to enhance organizational climates favorable to evidence-based chronic disease prevention. In comparison states, the study team will provide links to pre-existing sources of evidence-based information such as the Community Guide, Cancer Control P.L.A.N.E.T. (Plan, Link, Act, Network, with Evidence-based Tools), and Research to Practice. Pre- and post- evaluation measures will include the survey and record abstraction tool developed in phase one, qualitative interviews, and social network analyses.

### Dissemination strategies

In each dissemination state, a core group of chronic disease unit members and study team members will work together to identify, select, and refine dissemination strategies pertinent to the work unit’s situations, priority topics, and broader agency and state government contexts. The purpose is to enhance capacity of state-level public health practitioners and work units to plan, promote, and evaluate local and statewide implementation of EBDM and EBPPs for the prevention of chronic diseases. Dissemination strategies will emphasize electronic modes of knowledge transfer and interactive knowledge exchange among each state’s core group. Knowledge transfer and exchange strategies will be informed by lessons learned from Canadian research with the help of the Canadian consulting investigators [44,55-59]. Key principles for dissemination strategy selection are the strategy will build chronic disease prevention practitioner and work unit capacity for EBDM; the strategy will be sustainable by state health departments to maintain after this grant-funded study ends; and the strategy will be developed and applied through participatory engagement [48,60-62]. Content will be targeted to the chronic disease risk factor prevalence and disease burden in each state and priority topic areas selected by the chronic disease unit [63,64]. Priority topic areas may include tobacco control, obesity prevention, physical activity, nutrition, cancer screening, skin cancer prevention, or coordinated chronic disease prevention. The initial dissemination strategy in each of the six states will be a targeted multi-day in-person dissemination workshop [63]. Potential additional dissemination strategies include:

1. Targeted electronic messaging across sites choosing similar topic areas;

2. Online discussion groups across sites;

3. Webinars;

4. Providing links to pre-existing evidence sources for easy access;

5. Conducting specific evidence searches in response to state requests and teaching state staff how to do this themselves;

6. Electronically-delivered issue briefs that provide public health evidence in user-friendly, one to two page formats with a combination of statistical information and narrative examples, which public health practitioners can share with state agency leaders, elected officials, and private funders [65-67];

7. Technical assistance on how to document use of EBDM and EBPP implementation;

8. Strategies to foster agency support for evidence-based chronic disease prevention in partnership with public health practitioners. Examples include finding ways to help agency leaders prioritize EBPPs, finding ways to persuade supervisors to expect EBDM use by staff, developing feasible incentives in state health departments for application of EBDM, and incorporating EBPPs in contracts with local partners;

9. Strategies to embed EBDM knowledge acquisition into ongoing work unit processes such as new employee orientation, job descriptions, and performance reviews.

### State selection and recruitment

Figure 2 depicts the paired cluster randomized design for state selection. The paired design is the most appropriate for this study because it is not feasible for us to obtain more than 12 states (clusters) in total. By using state population as the main matching criterion, we will balance some important state-level factors (*e.g.*, state population is highly correlated (0.72) with chronic disease funding from the CDC) potentially affecting the trial outcomes. As a result, the between-cluster variation will be reduced, resulting in a gain in the statistical power [68,69]. Participating states and individuals will be aware of state status as dissemination or comparison; there is no blinding.

Based on our preliminary studies and values of ICC in the literature [32,44,70-74], we have estimated a range of effect sizes and ICCs. ICC estimates are the most difficult to obtain; we calculated a median ICC from similar studies and developed a range based on a 50% decrease and increase around the median (range 0.009 to 0.027). The sample size requirement is based on testing three hypotheses with a power of >90% and the overall type I error of 5% given six paired clusters (states). The null hypotheses involve the change in the scores from baseline of three outcomes— EBDM resources (awareness phase), supervisor support and expectations for EBDM use (adoption), and evaluation for maintenance of EBDM—in both the intervention and control arms (no change). Drawing from our previous work [32,44,74], the corresponding three alternative hypotheses for the change in scores in the intervention arm are 17%, 20%, 14%, higher for EBDM resources needed for awareness of the evidence, supervisory support and expectations for EBDM adoption/use, and evaluation for maintenance of EBDM, respectively. Following Donner [75] and Thompson [76], and our previous ability to obtain high response rates [32,74], we estimated the number of subjects needed in each state as 59 (total = 708). We calculated the number of subjects needed using calculated ICCs of 0.055 and 0.051 for EBDM resources and supervisory support, respectively, from the phase one national survey data, with a resulting number of participants needed in each state of 62 (total = 744). This assumes a 74% response rate (0.74 × 84). All individual chronic disease unit public health practitioners in participating states will be invited into the study (complete enumeration), and a purposive sample of coalition and local health department partners identified by the states will also be invited into the study.

Using IBM SPSS 20, eligible states will be de-identified, assigned random case numbers, and stratified by state population tertiles. Selection, pair-matching, and enrollment will be staggered by two pairs at a time over years two and three of the study for feasibility of conducting phase two activities. By the end of the staggered selection and enrollment, two state health departments will be randomly selected from each of the three state population strata and paired with the state closest in population. The principal investigator (last author) will invite chronic disease directors from each selected state health department to have their chronic disease units participate in the study and agree to be randomly assigned to dissemination or comparison conditions. If any state health department declines, the state next closest in population will be selected as a replacement match. After achieving a complete consented pair, the third author will de-identify states and randomly assign dissemination or comparison using the random case selection function in SPSS. After random assignment occurs, the principal investigator will communicate the results to the pair.

State eligibility among the 51 state health departments (50 states and the District of Columbia) for selection will be based on four criteria: an aggregate state EBDM capacity index factor score derived from the national survey, with five outlier low or high states excluded; a state cancer and chronic disease excess mortality index derived from archival data, with three outlier low burden states excluded; extent of EBDM training and technical assistance received by the principal investigator and teams, which excluded seven states with recent trainings; and availability of a logical pair-matched state, with three states excluded.

The two calculated indices for state eligibility criteria are described below. The EBDM capacity index will include five variables aggregated to states from individual respondents’ self-reported survey responses: resources for EBDM scale (eight items), organizational climate scale (five items), availability of EBDM skills scale (ten items), use of EBDM (a single item), and supervisor expectation of EBDM use (a single item). Individual scores will be aggregated to each state based on median scores. Confirmatory factor analysis will be used to examine the construct validity of each of the scales (*e.g.*, organizational climate). The few states with outlier high and low EBDM capacity will be excluded on the rationale of lack of need or lack of readiness for the study. The state chronic disease excess mortality and risk index will be derived from national mortality rates of lung cancer, breast cancer, cervical cancer, colorectal cancer, coronary heart disease, stroke, diabetes, and chronic obstructive pulmonary disease, and prevalence of adult tobacco use, physical inactivity, unhealthy diet, and obesity. States with outlier low excess mortality and risk will be excluded. Additionally, several states with recent extensive training and technical assistance support from our research center will also be excluded, and only the contiguous 48 states will be considered for eligibility due to travel costs.

In dissemination states, state health department cancer and other chronic disease prevention statewide partners will be recruited into the study, as well as key state level partners from statewide coalitions and other governmental and non-governmental organizations. Additional key local public health partners will be recruited as well, with the help of the chronic disease directors, comprehensive cancer program managers, and other practitioners. In each state, we anticipate an average of 20 participants in the state health department, 10 partners from non-profit organizations such as the American Cancer Society, and 30 local public health system participants, for an average of 70 per state. Additional state pairs will be enrolled in years two and three of the study and then randomized to active dissemination or comparison status, and partners identified with the help of the chronic disease directors and comprehensive cancer program managers.

### Evaluation for phase two

The evaluation of the dissemination strategies will track the knowledge transfer, exchange, and utilization as depicted in Figure 1 in combination with the dissemination stages of awareness, adoption, implementation, and maintenance [38,63]. The primary outcomes will be pre-post change scores in knowledge use and organizational supports for EBDM examined between dissemination and comparison states. Primary outcomes will be measured through individual and organizational level self-report survey items determined through phase one (see Table 2). Primary outcomes include: perceived resources for EBDM, resource use, supervisory support and expectations for EBDM use, knowledge transfer and exchange, evaluation maintenance, agency leadership support for EBDM, and self-reported use of EBDM. Data will be collected from individual state public health practitioners pre-initiation of dissemination activities in their respective work unit and 18 months post-initiation. Additionally, a secondary outcome, EBDM skill gaps pre-post, will be measured and examined across dissemination and comparison states. Following Phase one’s instrument tool, EBDM skill gaps are measured as the difference in importance and availability of specific skills required for EBDM. These gaps are measured at the individual practitioner level and will be aggregated to the state level for comparison between dissemination and comparison states.

Independent variables include participant characteristics, characteristics of the state health department, and state contextual variables such as rurality of the state and political affiliations of the governor and state legislatures. Quantitative data will come from three instruments: the national survey instrument, the archival record abstraction form, and a social network analysis instrument to be developed with input from the study international advisory group. Additional information will be gathered in qualitative interviews (face-to-face or by phone), recording project costs (*e.g.*, labor, supplies, equipment, travel), and process evaluation. Process indicators will also be measured to assess on an ongoing basis throughout phase two, variables such as staff time needed to develop and coordinate the trainings, participation rates across sites, how effectively partners are involved across sites, and how the state health department promotes EBPP implementation among local entities.

### Data analyses

From the cross-sectional phase one national survey data, confirmatory factor analyses will be conducted to test and refine the hypothesized dissemination stage variable groupings from observed data. Multilevel structural equation modeling will be conducted if needed. Descriptive statistics will be calculated for all variables, and chi-square and t-tests will be applied to compare subgroups of participants. Multivariate linear and logistic regression modeling will be conducted to test for hypothesized associations. For phase two, we will conduct bivariate analyses to explore associations and multivariate analyses within and across time allowing for adjustment. Multilevel regression modeling will be utilized for the analysis of pre-post change scores to account for variance across and within clusters.

Qualitative interview recordings from key informant interviews will be transcribed and the transcripts reviewed for completeness and accuracy. Each interview will then be coded by two coders [77]. This method will use the interview guide questions to establish major categories such as organizational factors. Matrices and tables will be created to facilitate comparisons between and within states [78].

### Study status

The study is currently ongoing. Some phase one activities have been completed (as documented above), including survey cognitive response testing, reliability test-retest data collection, and data collection of the national survey. The development of an abstract tool to record uptake of EBPPs from state health department program area plans and reports is close to completion.

Phase two, the cluster randomized trial, is in the planning phase. Planning is in progress with development of the state chronic disease excess mortality and risk index and a state EBDM capacity index for the 50 states and the District of Columbia. State selection criteria have been established. State pair-matched randomization procedures have been reviewed with a statistician and the full study advisory group and revised. An August 2013 in-person meeting furthered phase two planning.

## Discussion

This study has the potential to be innovative in several ways. This study will be among the first to provide the public health field with information about the facilitators and strategies that state level practitioners use in evidence based chronic disease prevention. Measures of dissemination among practitioners working in prevention of cancer and other chronic diseases are lacking [79-82]. This study will be among the first to develop, test, and utilize such measures. This study will apply dissemination lessons learned from Canada, a leader in knowledge transfer and exchange efforts internationally [36,44,47,59,83-85]. This study is among the first to apply Institutional Theory in conjunction with frameworks used in public health, specifically Diffusion of Innovations and a knowledge transfer and utilization framework. This study’s flexible participatory engagement approach in which enrolled dissemination states will choose the dissemination strategies that best fit their situation contributes to the external validity of the study findings [48]. The study may also promote greater collaboration between practitioners and researchers and help quicken the transfer of knowledge between researchers and practitioners. It is innovative to measure dissemination strategies to assess how best to promote sustainable and ongoing evidence-based practices.

The study has the potential for future large scale impact as it may identify effective ways to disseminate public health knowledge needed for EBDM processes in different contexts and help shorten the time between research evidence discovery and program application delivery. This study is also timely given the recent emphasis by the National Center for Chronic Disease Prevention and Health Promotion at CDC for states to use EBPPs in each program area funded by CDC and to do so through increasingly coordinated chronic disease prevention programming. Phase two dissemination strategies will help prepare practitioners to plan and evaluate evidence-based approaches to common risk factors, which is the target of coordinated approaches.

The study is subject to a few limitations. Our main limitations result from a relatively small number of pairs in the group randomized design and the limited pool for recruiting individuals who will be recruited for self-report data collection. Randomization here mainly reduces selection bias, because randomization of the small number of pairs without increased within group n size does not significantly improve statistical power [86]. Another limitation to our evaluation efforts is that study activities will be completed within the context of a dynamic ‘real world’ environment and will be complemented by the CDC’s existing and growing push for EBPPs, which will make it difficult to find pre-post differences between dissemination and comparison states. CDC now requires use of EBPPs when funding states for the major risk factor and chronic disease programs and provides varying degrees of technical assistance. However, with triangulated data collection efforts we increase the study’s ability to examine our multifaceted contribution. Even with the potential for large-scale impact, the nature of a state based approach has contextual challenges, including funding reductions and staff turnover. For example, the already tough funding climate for population-based non-clinical prevention programs in cancer and other chronic diseases has recently seen more reductions. Over half (56.9%) of the state health departments have CDC funding cuts for 2013 – 2014 in major chronic disease programs [87]. This study will seek to address the challenging funding climate by training staff on communicating prevention priorities to policymakers, making public health evidence available in ways that save staff time to access and digest, and providing technical assistance in grant writing and diversification of funding sources in the six dissemination states, with tools then made available to other states. In phase two, staff turnover will be monitored and managed via frequent communication with practitioners in the six dissemination states.

In conclusion, if cancer prevention and early detection programs and policies known to be effective were applied throughout the United States, an eventual one-third reduction in cancer mortality is feasible [88,89]. This study’s findings will further a small and growing body of knowledge on how best to support uptake of evidence-based approaches among public health practitioners.

## Competing interests

The authors declare that they have no competing interests.

## Authors’ contributions

Conceptualization and design: RCB, EAB, JK, EJ, KAS; Survey instrument development: RCB, EAB, JK, EJ, JKH, KAS, PA, LE, SS; Survey instrument testing: PA, LE, SS, AAH; Statistical support: AAH, RRJ; Writing: PA, SS, RCB; Manuscript content revisions: EAB, EJ, JK, JKH, KAS. All authors read and approved the final manuscript.

## Authors’ information

RCB: PhD, Professor and Co-Director, Prevention Research Center in St. Louis, Brown School; Division of Public Health Sciences and Siteman Cancer Center, Washington University School of Medicine; Washington University in St. Louis.

PA: PhD, MPH, RN, Assistant Research Professor, Prevention Research Center in St. Louis, Brown School, Washington University in St. Louis.

EAB: PhD, MPH, Professor and Co-Director, Prevention Research Center in St. Louis, College for Public Health & Social Justice, Saint Louis University.

MD: RN, PhD, Scientific Director, National Collaborating Centre for Methods and Tools and Health Evidence, McMaster University, Ontario, Canada.

LE: BA, Graduate Research Scholar, Prevention Research Center in St. Louis, Brown School, Washington University in St. Louis.

JKH: PhD, Assistant Professor, Brown School, Washington University in St. Louis.

AAH: MSc, Doctoral candidate at the Federal University of Parana, Department of Physical Education, Curitiba, Parana, Brazil; School of Health and Biosciences, Pontifícia Universidade Católica do Paraná, the Department of Physical Education; Visiting Scholar, Prevention Research Center in St. Louis, Brown School, Washington University in St. Louis.

RRJ: MSW, MPH, Statistical Data Analyst, Prevention Research Center in St. Louis, Brown School, Washington University in St. Louis.

EJ: PhD, CHES, Assistant Professor, School of Health Related Professions, University of Mississippi Medical Center.

JFK: PhD, Senior Scientific Lead for Population Health and Knowledge Management, Canadian Partnership Against Cancer.

SS: MSW/MPH, Prevention Research Center in St. Louis, Brown School, Washington University in St. Louis.

KAS: PhD, MPH, Associate Professor, Department of Epidemiology and Prevention Research Center in St. Louis, College for Public Health & Social Justice, Saint Louis University.
